# Heterocyclic-2-carboxylic Acid (3-Cyano-1,4-di-*N*-oxidequinoxalin-2-yl)amide Derivatives as Hits for the Development of Neglected Disease Drugs

**DOI:** 10.3390/molecules14062256

**Published:** 2009-06-22

**Authors:** Saioa Ancizu, Elsa Moreno, Enrique Torres, Asunción Burguete, Silvia Pérez-Silanes, Diego Benítez, Raquel Villar, Beatriz Solano, Adoración Marín, Ignacio Aldana, Hugo Cerecetto, Mercedes González, Antonio Monge

**Affiliations:** 1 Unidad en Investigación y Desarrollo de Medicamentos, Centro de Investigación en Farmacobiología Aplicada (CIFA), University of Navarra, C/Irunlarrea s/n, 31008 Pamplona, Spain; E-mails: sancizupere@alumni.unav.es (S.A.), emoreno4@alumni.unav.es (E.M); 2 Laboratorio de Química Orgánica, Facultad de Ciencias/Facultad de Química, Universidad de la República, Iguá 4225, 11400 Montevideo, Uruguay; E-mails: dbenitez@fq.edu.uy (D.B.), hcerecet@fq.edu.uy (H.C.)

**Keywords:** quinoxaline, neglected diseases, *Mycobacterium tuberculosis*, *Trypanosoma cruzi*

## Abstract

Neglected diseases represent a major health problem. It is estimated that one third of the world population is infected with tuberculosis (TB). Besides TB, Chagas disease, affects approximately 20 million people. Quinoxalines display great activities against TB and Chagas. Forty new quinoxaline 1,4-di-*N*-oxide derivatives have been prepared and tested against *M. tuberculosis* and *T. cruzi*. Carboxylic acid quinoxaline 1,4-di-*N*-oxides (CAQDOs) **5 **and **17** showed MIC values on the same order as the reference antituberculosis drug, rifampicin. Meanwhile, CAQDOs **12 **and **22** presented IC_50_ values in the same order as the anti-chagasic drug, nifurtimox.

## Introduction

**Scheme 1:**
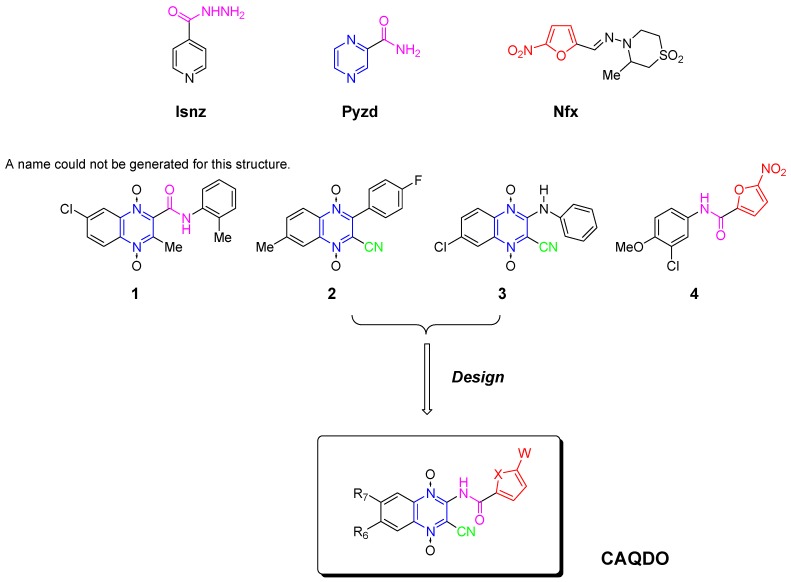
Design of new QDOs as potential drugs against neglected diseases.

*Mycobacterium tuberculosis* (*M. tuberculosis*), and to a lesser extent *M. bovis* and *M. africanum*, can cause a chronic and fatal condition in humans known as tuberculosis (TB). Until about 50 years ago, this disease was considered virtually incurable. The discovery of several active anti-TB agents heralded a new age of anti-TB chemotherapy. Therefore, TB was considered to be a curable disease. Unfortunately, in only a few years, it became apparent that the use of these drugs as single agents led to rapid drug resistance and treatment failures among a substantial number of patients. It was quickly realized, however, that the development of resistance could be forestalled or prevented through treatment with several active agents in a combination regimen. Of particular concern is the development of multi-drug-resistant forms of the disease (MDR-TB), defined as forms resistant to two or more of the front line anti-TB agents. These forms of the disease are most often fatal and are difficult and expensive to treat. It is estimated that one third of the world’s population is infected with TB, with about eight million new cases annually. Of these cases, 3.1 million die annually, more deaths than those caused by any other single infectious disease. TB is the leading killer of youths, women, and AIDS patients in the world [1,2]. HIV-infected patients have an elevated risk of tuberculosis, and such active infectious process may enhance HIV replication and increase the risk of death. It has been estimated that up to 50 million people are infected with drug-resistant forms of TB. Due to the fact that the current frontline therapy for TB consists of administering three different drugs (the antibiotic rifampicin, RIF, and the azaheterocycles isoniazid and pyrazinamide, Isnz and Pyzd, Scheme 1) over an extended period of time as well as the problems that arise due to MDR-TB, it is necessary to develop new, potent, fast-acting anti-tuberculosis drugs with low-toxicity profiles for treating drug resistant forms of the disease that can be given in conjunction with drugs used to treat HIV infections [3,4].

Besides TB, the parasitic diseases represent a major health problem in Third World countries. More specifically, Chagas disease, or American trypanosomiasis, caused by the protozoan *Trypanosoma cruzi* (*T. cruzi*), is the largest parasitic disease burden in the American continents. It affects approximately 20 million people from the southern United States to southern Chile. Even though the enforcement of public health programs towards vector elimination in some Latin American countries has decreased the incidence of new infections, the disease is still endemic in large areas. Every year, 21,000 people die from this parasitosis and over 200, 000 new cases arise [5]. Currently, there are only two clinically used drugs, nifurtimox (Nfx, Scheme 1) and benznidazole. Both are nitroheterocyclic compounds that possess important toxic effects and relative clinical efficacy; therefore, the pharmacotherapy of Chagas disease is very deficient and there is an urgent need for the development of safe and effective drugs [6].

Quinoxalines, including their fused-ring derivatives, display diverse pharmacological activities and more specifically, their 1,4-di-*N*-oxides have demonstrated excellent activities as antiviral, anticancer, antibacterial, and antiparasitic agents [7]. In this sense, our group published several studies in which the synthesis and biological evaluation of a large amount of quinoxaline 1,4-di-*N*-oxides (QDO) have been described. We have focused our recent efforts on the development of QDO with activities against the agents responsible of some of the well-known neglected diseases. We have found QDO with good *in vitro* selectivity against *M. tuberculosis* (i.e. parent compounds **1** and **2**, Scheme 1) [8,9,10,11,12,13,14,15,16] and against *T.cruzi* (i.e. parent compound **3**, Scheme 1) [17,18], and in both cases, recognizing some structural requirements for optimal activity [12,13,14,15,16,17,18,19,20].

Galactofuranose is an essential component of the mycobacterial cell wall, not found in man; UDP-galactofuranose is biosynthesized from UDP-galactopyranose using the enzyme UDP-galactose mutase (Glf). In 2004, Tangallapally *et al*. discovered that nitrofuryl derivatives have the requirements for optimum inhibition of Glf activity (i.e. compound **4**, Scheme 1) [4]. In addition, the nitrofuryl moiety is present in a large number of anti-*T. cruzi* agents acting via a nitroreduction process, generating redox cycling at different levels [21]. Based on these structural features, we have designed a new series of quinoxaline 1,4-di-*N*-oxide derivatives containing a nitrofuryl side chain as potential anti-neglected diseases agents. More specifically, we have designed new hybrid QDO with potential anti-tubercular activity combining some previous structural features, amide and cyano QDO substituents, and the heteroaryl retro-amide moieties. In order to determine the action of the 5-nitrofuryl moiety, we synthesized another analogue series by substituting this group by a 5-nitrothienyl one, and in order to determine the influence of the nitro group, another two series were designed, furyl and thienyl side chains (Table 1). Moreover, the same designed compounds could also act as hybrid potential anti-*T. cruzi* agents because they are QDO that have maintained the 3-cyano and 2-NH, as retro-amide moieties, with the extra-heteroaryl substituents (5-nitrofuryl, 5-nitrothienyl, furyl, and thienyl groups).

## Results and Discussion

**Scheme 2:**
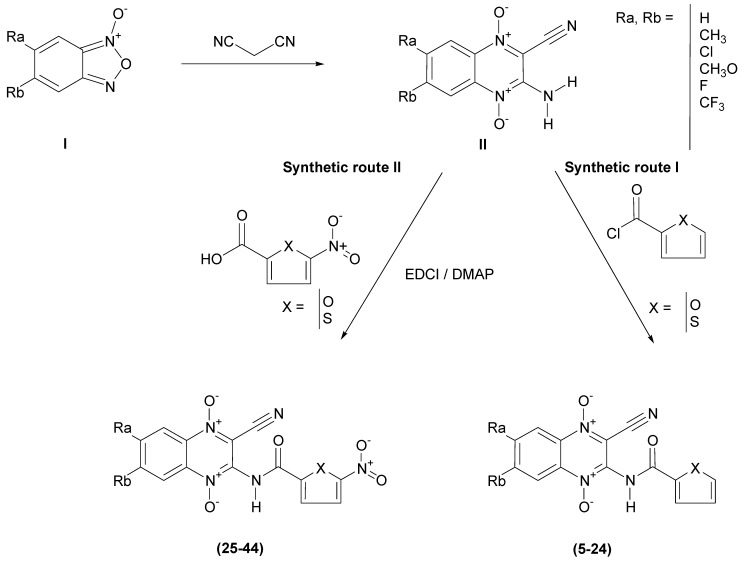
Synthetic route to heterocyclic-2-carboxylic acid (3-cyano-1,4-di-*N*-oxidequinoxalin-2-yl)-amide derivatives **5-44**.

We have prepared forty new heterocyclic-2-carboxylic acid (3-cyano-1,4-di-*N*-oxidequinoxalin-2-yl)-amide derivatives (CAQDOs). The benzofuroxane starting compounds, (BFX, **I**, Scheme 2), have been prepared using previously described methods [11,22]. The 3-amine-1,4-di-*N*-oxide quinoxaline-2-carbonitrile derivatives (cyanoamines, **II**) were obtained by the Beirut reaction from the corresponding BFX, with malononitrile using *N,N*-dimethylformamide (DMF) as solvent and triethylamine as catalyst [23]. Finally, the new CAQDOs were synthesized using two different routes, I and II (Scheme 2) [4,24]. Furyl and thienyl derivatives **5-24** were obtained by reaction between intermediates **II** with an excess of the corresponding, commercially available, heteroaryl-2-carbonyl chloride. Another synthetic route was optimized for the synthesis of 5-nitrofuryl and 5-nitrothienyl derivatives **25-44**. In these cases, the reaction was carried out by condensation between intermediates II and the corresponding carboxylic acid, 5-nitrofuryl and 5-nitrothienyl carboxylic acid, activated in the presence of EDCI and DMAP and DMF as solvent. 

In order to carry out an in depth QSAR study, CAQDO were un-substituted or substituted in positions 6 and 7 by chloro- or fluoro- or trifluoromethyl-moiety as electron-withdrawing groups and by methyl- or methoxy-moiety as electron-releasing groups. When the new CAQDOs were prepared from monosubstituted-BFX, a mixture of positional isomers was obtained. Generally, it could be observed that the 6-substituted isomer prevailed over the 7-substituted isomer [25]. When the substituent was a methoxy moiety, a regio-specific reaction was achieved because only 6-isomer was isolated. In the other case, when 5-trifluoromethyl-BFX was reacted, the two CAQDO-isomers were obtained in similar proportions and separated by chromatography.

The new developed CAQDOs were subjected to the following set of tests: i) determination of the MICs, in μg/mL, against *M. tuberculosis* H37Rv strain and, ii) determination of the percentage of growth inhibition, at 25 μM, and IC_50_ values, in μM, against *T. cruzi* Tulahuen 2 strain (Table 1).

With regard to the anti-*M. tuberculosis* evaluations, the CAQDO **5** and **17** were identified as the most active derivatives against H37Rv strain, with MIC values on the same order as the reference compound, RIF (Table 1). Some structure-activity relationships could be established; in general, thienyl-derivatives are more active than furyl derivatives (cf. the anti-*M. tuberculosis* activities of **16** and **6**, **19** and **9**, **23** and **13**, **24** and **14**, **38** and **28**, **43** and **33**, or **44** and **34**), whereas the effect of the 5-nitro substitution is clear only for the thienyl series (cf. the anti-*M. tuberculosis* activities of **35** and **15**, **38** and **18**, **43** and **23**, or **44** and **24**), with the 5-nitrothienyl-derivatives being more active than the un-substituted ones. For this biological activity we were unable to find relationships between this and the electronic characteristics of benzo-substituent on the quinoxaline heterocycle. However, it could be pointed out, bearing in mind derivatives **29-32**, that the mono-halogen substitution produce compounds more actives than the di-halogen substituted ones furthermore chlorine-substitution is better for the activity than fluorine-substitution. Considering the couple of derivatives **13** and **14**, **23** and **24**, **33** and **34**, and **43** and **44** the 7-trifluoromethyl-substitution produces more active compounds.

With regard to the anti-*T. cruzi* evaluations, the difluoro substituted CAQDOs **12** and **22** were identified as the most active derivatives against the Tulahuen 2 strain, with IC_50_ values on the same order as the reference compound, Nfx. Moreover, compound **22** was found more active than the parent compound **3** (Scheme 1, Table 1). Similar to *M. tuberculosis*, the thienyl-derivatives are more active than furyl derivatives (compare anti-*T. cruzi* activities of **22** and **12**), unlike in the case of *M. tuberculosis*, in which the influence of the 5-nitro substitution is clear, with the 5-nitro-substituted derivatives being less active than the un-substituted derivatives (compare anti-*T. cruzi* activities of **42** and **22**). In *T. cruzi* findings, some relationships between the electronic characteristics of benzo-substituent, on the quinoxaline heterocycle, and the activity could be established; for example, when the electron-withdrawing property increases, the activity increases (compare activity of compound **10** with **8** or **7**, **12** and **10**, **22** and **21**, or **40** and **39**). The hybridization process, pharmacophore quinoxaline dioxide plus pharmacophore nitrofurane, does not produce active compounds.

**Table 1:** Biological characterization of the forty new quinoxaline 1,4-di-*N*-oxides. 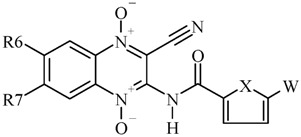

Cpd.	X	W	R7	R6	MIC^a^(μg/mL)	%GI^b^
**5**	O	H	H	H	0.977	3.1
**6**	O	H	H	CH_3_	8.843	11.6
**7**	O	H	CH_3_	CH_3_	NT^c^	13.1
**8**	O	H	H	OCH_3_	NT	9.5
**9**	O	H	H	Cl	15.361	NT
**10**	O	H	Cl	Cl	NT	31.6
**11**	O	H	H	F	NT	19.8
**12**	O	H	F	F	4.700	53.1 (IC_50_ = 19.2)
**13**	O	H	CF_3_	H	17.903	NT
**14**	O	H	H	CF_3_	24.220	NT
**15**	S	H	H	H	5.381	19.0
**16**	S	H	H	CH_3_	2.082	16.4
**17**	S	H	CH_3_	CH_3_	1.190	13.5
**18**	S	H	H	OCH_3_	48.909	15.1
**19**	S	H	H	Cl	2.470	NT
**20**	S	H	Cl	Cl	13.612	NT
**21**	S	H	H	F	1.474	27.5
**22**	S	H	F	F	NT	92.4 (IC_50_ = 10.8)
**23**	S	H	CF_3_	H	4.576	NT
**24**	S	H	H	CF_3_	9.177	NT
**25**	O	NO_2_	H	H	NT	0.7
**26**	O	NO_2_	H	CH_3_	NT	0.0
**27**	O	NO_2_	CH_3_	CH_3_	NT	0.0
**28**	O	NO_2_	H	OCH_3_	14.493	NT
**29**	O	NO_2_	H	Cl	5.811	NT
**30**	O	NO_2_	Cl	Cl	13.573	NT
**31**	O	NO_2_	H	F	21.675	NT
**32**	O	NO_2_	F	F	22.161	NT
**33**	O	NO_2_	CF_3_	H	12.816	NT
**34**	O	NO_2_	H	CF_3_	14.408	NT
**35**	S	NO_2_	H	H	2.875	0.0
**36**	S	NO_2_	H	CH_3_	NT	11.4
**37**	S	NO_2_	CH_3_	CH_3_	NT	36.4
**38**	S	NO_2_	H	OCH_3_	9.538	NT
**39**	S	NO_2_	H	Cl	NT	15.0
**40**	S	NO_2_	Cl	Cl	NT	29.8
**41**	S	NO_2_	H	F	NT	2.5
**42**	S	NO_2_	F	F	NT	9.2
**43**	S	NO_2_	CF_3_	H	1.695	NT
**44**	S	NO_2_	H	CF_3_	6.001	NT
**3**	-	-	-	-	-	63.0^d^
**RIF**	-	-	-	-	0.125	-
**Nfx**	-	-	-	-	-	100.0 (7.7)

^a ^Minimum inhibitory concentration against *M. tuberculosis* H37Rv. ^b ^Percentage of growth inhibition at 25 μM doses in *T. cruzi* Tulahuen 2 strain*.* ^c ^NT: Not tested. ^d ^From reference [17].

## Experimental

### General

All of the synthesized compounds were chemically characterized by thin layer chromatography (TLC), infrared (IR), proton nuclear magnetic resonance (^1^H-NMR), mass spectra (MS) and elemental microanalyses (CHN). Alugram SIL G/UV254 (Layer: 0.2 mm) (Macherey-Nagel GmbH & Co. KG., Düren, Germany) was used for TLC and Silica gel 60 (0.040-0.063 mm, Merck) was used for Flash Column Chromatography. The ^1^H-NMR spectra were recorded on a Bruker 400 Ultrashield instrument (400 MHz), using TMS as internal standard and with DMSO-d_6_ as solvent; the chemical shifts are reported in ppm (δ) and coupling constants (*J*) values are given in Hertz (Hz). Signal multiplicities are represented by: s (singlet), bs (broad singlet), d (doublet), t (triplet), q (quadruplet), dd (double doublet) and m (multiplet). The IR spectra were recorded on a Nicolet Nexus FTIR (Thermo, Madison, USA) in KBr pellets. Elemental microanalyses were obtained on a CHN-900 Elemental Analyzer (Leco, Tres Cantos, Spain) from vacuum-dried samples. The analytical results for C, H and N, were within ± 0.5 of the theoretical values. Chemicals were purchased from Panreac Química S.A. (Barcelona, Spain), Sigma-Aldrich Química, S.A. (Alcobendas, Spain), Acros Organics (Janssen Pharmaceutical, Geel, Belgium) and Lancaster (Bischheim-Strasbourg, France).

### General procedure for the synthesis of cyanoamines **II**

Malononitrile (18.0 mmol) was added to a solution of the appropriate benzofuroxane (**I**, 15.0 mmol) in DMF (10 mL). The mixture was allowed to stand at 0 °C. Triethylamine was added dropwise (1.5 mL), and the reaction mixture was stirred at room temperature in darkness for 1-3 days. The precipitate was filtered off and washed by adding diethyl ether affording the target compound. The obtained red solid was used in the next step without further purification.

### General procedure for the synthesis of furan-2-carboxylic acid (3-cyano-1,4-di-N-oxide-quinoxalin-2-yl)-amide derivatives and thiophene-2-carboxylic acid (3-cyano-1,4-N-oxide-quinoxalin-2-yl)-amide derivatives **5-24**

The corresponding 3-amino-1,4-di-*N*-oxide-quinoxaline-2-carbonitrile **II** (2 mmol) is dissolved in acetonitrile (100 mL) and triethylamine (0.4 mL) was added at room temperature under stirring and anhydrous conditions. After cooling the reaction mixture with an ice bath, 2-furoyl chloride or 2-tiophenecarbonyl chloride (2.2 mmol) are added. The reaction mixture is stirred for 9 h at room temperature. The obtained solid is filtered and EtOAc (400 mL) added to the filtrate. The organic phase is extracted, first with HCl 10% and then with water. The organic phase is dried with anhydrous Na_2_SO_4_ and filtered. The solvent is removed *in vacuo* and the resulting residue is precipitated with diethyl ether, and then filtered in order to obtain a yellow-orange solid.

*Furan-2-carboxylic acid (3-cyano-1,4-di-N-oxidequinoxalin-2-yl)amide* (**5**). Yield 64.4 %; ^1^H-NMR δ ppm: 11.43 (bs, 1H, NH); 8.53-8.48 (m, 2H, H_8_+H_5_); 8.13-8.01 (m, 3H, H_6_+H_7_+H_5’_); 7.60 (d, 1H, H_3´_, *J*_3´-4´ _= 3.5 Hz); 6.81 (dd, 1H, H_4´_, *J*_4´-5 ´_ = 1.7 Hz); IR ν cm^-1^: 3,237 (m, NH); 2,236 (w, C≡N); 1,686 (s, C=O); 1,335 (s, N^+^O^-^); Anal. Calc. for C_14_H_8_N_4_O_4_: C: 56.76%; H: 2.72%; N: 18.91%. Found: C: 56.73%; H: 3.13%; N: 18.93%.

*Furan-2-carboxylic acid (3-cyano-1,4-di-N-oxide-6-methylquinoxalin-2-yl)amide* (**6**). Yield 63.0 %; ^1^H-NMR δ ppm: 11.37 (bs, 1H, NH); 8.42 (d, 1H, H_8_, *J*_8-7 _= 8.8 Hz); 8.31 (d, 1H, H_5_, *J*_5-7 _= 1.3 Hz); 8.10 (d, 1H, H_5´_, *J*_5´-4´ _= 1.7 Hz); 7.95 (dd, 1H, H_ 7_); 7.69 (d, 1H, H_3´_, *J*_3´-4´ _= 3.6 Hz), 6.81 (dd, 1H, H_4´_), 2.61 (s, 3H, CH_3_); IR ν cm^-1^: 3,114 (m, NH); 2,238 (w, C≡N); 1,692 (s, C=O); 1,325 (s, N^+^O^‑^); Anal. Calc. for C_15_H_10_N_4_O_4_: C: 58.07%; H: 3.25%; N: 18.06%. Found: C: 57.95%; H: 3.27%; N: 18.43%.

*Furan-2-carboxylic acid (3-cyano-1,4-di-N-oxide-6,7-dimethylquinoxalin-2-yl)amide* (**7**). Yield 75.3 %; ^1^H-NMR δ ppm: 11.33 (bs, 1H, NH); 8.31 (s, 1H, H_8_); 8.28 (s, 1H, H_5 _); 8.09 (d, 1H, H_5´_, *J*_5´-4´ _= 1.5 Hz); 7.68 (d, 1H, H_3´_, *J*_3´-4´ _= 3.5 Hz); 6.80 (dd, 1H; H_4´_); 2.54-2.52 (m, 6H, 2xCH_3_); IR ν cm^-1^: 3,285 (w, NH); 2,236 (w, C≡N); 1,709 (s, C=O); 1,324 (s, N^+^O^-^); Anal. Calc. for C_16_H_12_N_4_O_4_: C: 59.26%; H: 3.73%; N: 17.28%. Found: C: 59.33%; H: 3.77%; N: 17.30%.

*Furan-2-carboxylic acid (3-cyano-1,4-di-N-oxide-6-methoxyquinoxalin-2-yl)amide* (**8**). Yield 50.5 %; ^1^H-NMR δ ppm: 11.31 (bs, 1H, NH); 8.44 (d, 1H, H_8_, *J*_8-7 _= 9.4 Hz); 8.09 (s, 1H, H_5_); 7.78 (d, 1H, H_5´_, *J*_5´-4´ _= 2.4 Hz); 7.73 (d, 1H, H_7_); 7.67 (d, 1H, H_3´_, *J*_3´-4’ _= 3.10 Hz), 6.80 (dd, 1H, H_4’_); 4.03 (s, 3H, OCH_3_); IR ν cm^-1^: 3,298 (m, NH); 2,241 (w, C≡N); 1,698 (s, C=O); 1,321 (s, N^+^O^-^); Anal. Calc. for C_15_H_10_N_4_O_5_: C: 55.22%; H: 3.09%; N: 17.17%. Found: C: 54.87%; H: 3.09%; N: 16.79%.

*Furan-2-carboxylic acid (3-cyano-6-chloro-1,4-di-N-oxidequinoxalin-2-yl)amide* (**9**). Yield 6.1 %; ^1^H-NMR δ ppm: 11.47 (bs, 1H, NH); 8.51-8.50 (m, 2H, H_8_+H_5_); 8.14 (dd, 1H, H_7_, *J*_7-8 _= 9.1 Hz, *J*_7-5 _= 2.3 Hz); 8.10 (d, 1H, H_5´_, *J*_5´-4 ´_ = 1.7 Hz); 7.70 (d, 1H, H_3´_, *J*_3´-4´ _= 3.6 Hz); 6.81 (dd, 1H, H_4´_, *J*_4´-3’ _= 3.6 Hz, *J*_4´-5’ _= 1.7 Hz); IR ν cm^-1^: 3,288 (m, NH); 2,241 (w, C≡N); 1,692 (s, C=O); 1,320 (s, N^+^O^-^); Anal Calc. for C_14_H_7_ClN_4_O_4_: C: 50.58%; H: 2.13%; N: 16.94%. Found: 50.91%; H: 2.23%; N: 16.69%.

*Furan-2-carboxylic acid (3-cyano-6,7-dichloro-1,4-di-N-oxidequinoxalin-2-yl)amide* (**10**). Yield 51.0 %; ^1^H-NMR δ ppm: 11.56 (bs, 1H, NH); 8.72 (s, 2H, H_8_+H_5_); 8.11 (dd, 1H, H_5´_, *J*_5´-4´ _= 1.7 Hz, *J*_5´-3´ _= 0.7 Hz); 7.72 (d, 1H, H_3´_, *J*_3´-4´ _= 3.6 Hz); 6.81 (dd, 1H, H_4_); IR ν cm^-1^: 3,275 (m, NH); 2,232 (w, C≡N); 1,702 (s, C=O); 1,336 (s, N^+^O^-^); Anal. Calc. for C_14_H_6_Cl_2_N_4_O_4_: C: 45.04%; H: 1.61%; N: 15.01%. Found: C: 45.34%; H: 1.75%; N: 15.18%.

*Furan-2-carboxylic acid (3-cyano-1,4-di-N-oxide-6-fluoroquinoxalin-2-yl)amide* (**11**). Yield 57.8 %; ^1^H-NMR δ ppm: 11.44 (bs, 1H, NH); 8.59 (dd, 1H, H_8_, *J*_8-F _= 5.0 Hz); 8.30 (dd, 1H, H_5_, *J*_5‑F _= 8.6 Hz, *J*_5-7 _= 2.7 Hz); 8.10 (d, 1H, H_5´_, *J*_5´-4´ _= 1.5 Hz); 8.03 (ddd, 1H, H_7_, *J*_7-F _= 7.9 Hz); 7.70 (d, 1H, H_3´_, *J*_3´-4´_ = 3.6 Hz); 6.81 (dd, 1H, H_4’_); IR ν cm^-1^: 3,263 (m, NH); 2,239 (w, C≡N); 1,681 (s, C=O); 1,328 (s, N^+^O^-^); 1,242 and 1,171 (m, Ar-F); Anal. Calc. for C_14_H_7_FN_4_O_4_: C: 53.51%; H: 2.25%; N: 17.83%. Found: C: 53.06%; H: 2.27%; N: 17.86%.

*Furan-2-carboxylic acid (3-cyano-6,7-difluoro-1,4-di-N-oxidequinoxalin-2-yl)amide* (**12**). Yield 66.1 %; ^1^H-NMR δ ppm: 11.50 (bs, 1H, NH); 8.65-8.58 (m, 2H, H_8_+H_5_); 8.10 (dd, 1H, H_5´_, *J*_5´‑4´_ = 1.6 Hz, *J*_5´-3´ _= 0.6 Hz); 7.71 (dd, 1H, H_3´_, *J*_3´-4´ _= 3.7 Hz); 6.81 (dd, 1H, H_4´_); IR ν cm^-1^: 3,215 (m, NH); 2,236 (w, C≡N); 1,689 (s, C=O); 1,340 (s, N^+^O^-^); 1,270 and 1,181 (m, Ar-F); Anal. Calc. for C_14_H_6_F_2_N_4_O_4_: C: 50.61%; H:1.82%; N: 16.86%. Found: C: 50.37%; H: 1.98%; N: 16.67%.

*Furan-2-carboxylic acid (3-cyano-1,4-di-N-oxide-7-trifluoromethylquinoxalin-2-yl)amide* (**13**). Yield 78.1 %; ^1^H-NMR δ ppm: 12.06 (bs, 1H, NH); 8.69 (s, 1H, H_8 _); 8.64 (d, 1H, H_5_, *J*_5-6 _= 9.0 Hz); 8.37 (d, 1H, H_6 _); 8.07 (d, 1H, H_5´_, *J*_5´-4´ _= 1.7 Hz); 7.66 (d, 1H, H_3´_); 6.79 (d, 1H, H_4´_, *J*_3´-4´ _= 3.5 Hz); IR ν cm^-1^: 3,279 (m, NH); 2,244 (w, C≡N); 1,688 (s, C=O); 1,325 (s, N^+^O^-^); 1,137 (s, CF_3_); Anal. Calc. for C_15_H_7_F_3_N_4_O_4_: C: 49.46%; H: 1.96%; N: 15.38%. Found: C: 49.18%; H: 2.02%; N: 15.31%.

*Furan-2-carboxylic acid (3-cyano-1,4-di-N-oxide-6-trifluoromethylquinoxalin-2-yl)amide* (**14**). Yield 31.1 %; ^1^H-NMR δ ppm: 12.06 (bs, 1H, NH); 8.72 (d, 1H, H_5_, *J*_5-7_ = 1.7 Hz); 8.66 (d, 1H, H_8_, *J*_8-7_ = 9.0 Hz); 8.30 (dd, 1H, H_7_, *J*_7-8 _= 9.0 Hz, *J*_7-5_ = 1.7 Hz); 8.09 (dd, 1H, H_5´_, *J*_5´-4´ _= 1.7 Hz, *J*_5´-3´_ = 0.7 Hz); 7.69 (dd, 1H, H_3´_, *J*_3´-4´_ = 3.6 Hz); 6.80 (dd, 1H, H_4´_); IR ν cm^-1^: 3,263 (m, NH); 2,232 (w, C≡N); 1,701 (s, C=O); 1,341 (s, N^+^O^-^); 1,132 (s, CF_3_); Anal. Calc. for C_15_H_7_F_3_N_4_O_4_: C: 49.46%; H: 1.96%; N: 15.38%. Found: C: 49.59%; H: 1.89%; N: 15.09%.

*Thiophene-2-carboxylic acid (3-cyano-1,4-di-N-oxidequinoxalin-2-yl)amide* (**15**). Yield 33.3 %; ^1^H- NMR δ ppm: 11.65 (bs, 1H, NH); 8.54 (dd, 1H, H_8_, *J_8-7 _*= 8.6 Hz, *J_8-6 _*= 0.7 Hz); 8.50 (dd, 1H, H_5_, *J_5-6 _*= 8.6 Hz, *J_5-7 _*= 0.8 Hz); 8.30 (dd, 1H, H_5’_, *J*_5’-4´_ = 3.7 Hz, *J*_5´-3’ _= 0.9 Hz); 8.12 (td, 1H, *J_6-7 _*= 7.1 Hz, H6); 8.10-8.00 (m, 2H, H_7 _, H_3’_); 7.32 (dd, 1H, H_4´_, *J*_4´-3’ _= 4.9 Hz, *J*_4’-5’ _= 3.9 Hz); IR ν cm^-1^: 3,231 (m, NH); 2,232 (w, C≡N); 1,681 (s, C=O); 1,326 (s, N^+^O^-^); Anal. Calc. for C_14_H_8_N_4_O_3_S: C: 53.85%; H: 2.56%; N: 17.95%. Found: C: 53.88%; H: 2.82%; N: 18.35%. 

*Thiophene-2-carboxylic acid (3-cyano-1,4-di-N-oxide-6-methylquinoxalin-2-yl)amide* (**16**). Yield 1.2 %; ^1^H-NMR δ ppm: 11.62 (bs, 1H, NH); 8.39 (d, H_8_, 1H, *J*_8-7 _= 8.8 Hz); 8.35 (s, 1H, H_5_); 8.30 (m, 1H, H_5’_); 8.05 (d, 1H, H_3’_, *J_3’-4 ’_* = 4.9 Hz); 7.88 (d, 1H, H_7_); 7.33-7.31 (m, 1H, H_4’_); 2.66 (s, 3H, CH_3_); IR ν cm^-1^: 3,276 (w, NH); 2,225 (w, C≡N); 1,664 (s, C=O); 1,329 (s, N^+^O^-^); Anal. Calc. for C_15_H_10_N_4_O_3_S: C: 55.22%; H: 3.07%; N: 17.18%. Found: C: 55.01%; H: 3.17%; N: 17.39%.

*Thiophene-2-carboxylic acid (3-cyano-6,7-dimethyl-1,4-di-N-oxidequinoxalin-2-yl)amide* (**17**). Yield 1.4 %; ^1^H-NMR δ ppm: 11.59 (bs, 1H, NH); 8.32 (s, 1H, H_5’_); 8.28 (s, 2H, H_8_+H_5_); 8.03 (s, 1H, H_3’_); 7.31 (s, 1H, H_4’_); 2.6-2.4 (m, 6H, 2xCH_3_); IR ν cm^-1^: 3,206 (m, NH); 2,225 (w, C≡N); 1,667 (s, C=O); 1,327 (s, N^+^O^-^); Anal. Calc. for C_16_H_12_N_4_O_3_S: C: 56.47%; H: 3.53%; N: 16.47%. Found: C: 56.72%; H: 3.64%; N: 16.72%.

*Thiophene-2-carboxylic acid (3-cyano-1,4-di-N-oxide-6-methoxyquinoxalin-2-yl)amide* (**18**). Yield 41.1 %; ^1^H-NMR δ ppm: 11.53 (bs, 1H, NH); 8.46 (d, 1H, H_8_, *J_8-7 _*= 9.4 Hz); 8.28 (dd, 1H, H_5’_, *J_5’-4’ _*= 3.8 Hz, *J_5’-3’ _*= 0.9 Hz); 8.05 (dd, 1H, H_3’_, *J_3’-4’_* = 3.8 Hz); 7.79 (d, 1H, H_5_, *J_5-7 _*= 2.7 Hz); 7.73 (dd, 1H, H_7_); 7.32 (dd, 1H, H_4’_); 4.03 (s, 3H, OCH_3_); IR ν cm^-1^: 3,276 (m, NH); 2,232 (w, C≡N); 1,673 (s, C=O); 1,320 (s, N^+^O^-^); Anal. Calc. for C_15_H_10_N_4_O_4_S: C: 52.63%; H: 2.93%; N: 16.37%. Found: C: 52.49%; H: 3.06%; N: 16.35%.

*Thiophene-2-carboxylic acid (6-chloro-3-cyano-1,4-di-N-oxidequinoxalin-2-yl)amide* (**19**). Yield 33.2 %; ^1^H-NMR δ ppm: 11.66 (bs, 1H, NH); 8.54-8.48 (m, 2H, H_8 _+H_5_); 8.30 (dd, 1H, H_5’_, *J_5’‑4’_* = 3.8 Hz, *J_5’-3’_* = 0.8 Hz); 8.15 (dd, 1H, H_7_, *J_7-8 _*= 9.30 Hz, *J_7-5 _*= 2.2 Hz,); 8.06 (dd, 1H, H_3’_, *J_3’-4’ _*= 5.0 Hz); 7.32 (t, 1H, H_4’_); IR ν cm^-1^: 3,295 (m, NH); 2,251 (w, C≡N); 1,671 (s, C=O); 1,323 (s, N^+^O^-^); Anal. Calc. for C_14_H_7_ClN_4_O_3_S: C: 48.48%; H: 2.02%; N: 16.16%. Found: C: 48.58%; H: 2.16%; N: 16.15%.

*Thiophene-2-carboxylic acid (3-cyano-6,7-dichloro-1,4-di-N-oxidequinoxalin-2-yl)amide* (**20**). Yield 19.4 %; ^1^H-NMR δ ppm: 8.66 (s, 1H, H_8 _); 8.64 (s, 1H, H_5_); 8.13 (s, 1H, H_5’_); 7.96 (d, 1H, H_3’, _*J_3’-4’ _=* 4.7 Hz) 7.32 (dd, 1H, H_4’, _*J_4’-5’ _*= 3.9 Hz,); IR KBr (trans): 3,263 (m, NH); 2,238 (w, C≡N); 1,668 (s, C=O); 1,335 (s, N^+^O^-^); Anal. Calc. for C_14_H_6_Cl_2_N_4_O_3_S: C: 44.09%; H: 1.57%; N: 14.70%. Found: C: 43.83%; H: 1.67%; N: 14.70%.

*Thiophene-2-carboxylic acid (3-cyano-1,4-di-N-oxide-6-fluoroquinoxalin-2-yl)amide* (**21**). Yield 46.0 %; ^1^H-NMR δ ppm: 11.63 (bs, 1H, NH); 8.60 (dd, 1H, H_8_, *J_8-7 _*= 9.5 Hz, *J_8-F _*= 4.9 Hz,); 8.31-8.27 (m, 2H, H_5_+H_5´_); 8.06-8.01 (m, 2H, H_7 _+H_3´_); 7.32 (t, 1H, H_4’_); IR ν cm^-1^: 3,263 (m, NH); 2,226 (w, C≡N); 1,665 (s, C=O); 1,327 (s, N^+^O^-^); Anal. Calc. for C_14_H_7_FN_4_O_3_S: C: 50.91%; H: 2.12%; N: 16.97%. Found: C: 51.18%; H: 2.24%; N: 17.06%.

*Thiophene-2-carboxylic acid (3-cyano-6,7-difluoro-1,4-di-N-oxidequinoxalin-2-yl)amide* (**22**). Yield 69.1 %; ^1^H-NMR δ ppm: 11.74 (bs, 1H, NH); 8.70-8.50 (m, 2H, H_8 _+H_5_); 8.25-8.35 (m, 1H, H_5’_); 8.07 (d, 1H, H_3’_, *J_3´-4´_* = 4.9 Hz); 7.34-7.31(m, 1H, H_4’_); IR ν cm^-1^: 3,238 (m, NH); 2,238 (w, C≡N); 1,673 (s, C=O); 1,341 (s, N^+^O^-^); Anal. Calc. for C_14_H_6_F_2_N_4_O_3_S: C: 48.27%; H: 1.72%; N: 16.01%. Found: C: 48.47%; H: 1.80%; N: 16.18%.

*Thiophene-2-carboxylic acid (3-cyano-7-trifluoromethyl-1,4-di-N-oxidequinoxalin-2-yl)amide* (**23**)**.** Yield 11.7 %; ^1^H-NMR δ ppm: 11.85 (bs, 1H, NH); 8.75 (d, 1H, H_8, _*J_8-6 _=* 0.75 Hz); 8.65 (d, 1H, H_5, _*J_5-6 _=* 9.0 Hz); 8.39 (dd, 1H, H_6, _*J_6-5 _=* 9.1 Hz, *J_6-8 _=* 1.8 Hz); 8.27 (dd, 1H, H_5’, _*J_5’-4’ _*= 3.8 Hz, *J_5’-3’ _*= 1.1 Hz); 8.05 (dd, 1H, H_3’, _*J_3’-4’ _=* 5.0 Hz); 7.31 (dd, 1H, H_4’_); IR KBr (trans): 3,263 (w, NH); 2,238 (w, C≡N); 1,668 (s, C=O); 1,335 (s, N^+^O^-^); Anal. Calc. for C_15_H_7_F_3_N_4_O_3_S: C: 47.37%; H: 1.84%; N: 14.74%. Found: C: 47.67%; H: 1.77%; N: 14.80%.

*Thiophene-2-carboxylic acid (3-cyano-6-trifluoromethyl-1,4-di-N-oxidequinoxalin-2-yl)amide* (**24**)*.* Yield 12.8 %; ^1^H-NMR δ ppm: 11.83 (bs, 1H, NH); 8.74 (s, 1H, H_5_); 8.68 (d, 1H, H_8, _*J_8‑7 _=* 9.24 Hz); 8.35-8.28 (m, 2H, H_7_, H_5’_); 8.06 (dd, 1H, H_3’, _*J_3’-4’ _=* 5.0 Hz, *J_3’- 5’ _=* 1.0 Hz); 7.33-7.31 (m, 1H, H_4’_); IR KBr (trans): 3,270 (w, NH); 2,226 (w, C≡N); 1,679 (s, C=O); 1,339 (s, N^+^O^-^); Anal. Calc. for C_15_H_7_F_3_N_4_O_3_S: C: 47.37%; H: 1.84%; N: 14.74%. Found: C: 47.11%; H: 1.74%; N: 14.64%.

### General procedure for the synthesis of 5-nitrofuran-2-carboxylic acid (3-cyano-1,4-di-N-oxide-quinoxalin-2-yl)amide derivatives and 5-nitrothiophene-2-carboxylic acid (3-cyano-1,4-N-oxide-quinoxalin-2-yl)amide derivatives **25-44**

To a solution of the corresponding 3-amino-1,4-di-*N*-oxide-quinoxaline-2-carbonitrile **II** (2 mmol) in DMF (5 mL) are added 5-nitrothiophene-2-carboxylic acid or 5-nitrofuran-2-carboxylic acid (3 mmol). The reaction mixture is then gently stirred at room temperature under anhydrous conditions. Continuing with the synthesis, *N*-(3-dimethylaminopropyl)-*N’*-ethylcarbodiimide hydrochloride (EDCI, 6 mmol) are added, and the color changes to dark-red. When the dissolution is completed, 4-dimethyl-aminopyridine (DMAP, 6 or 7 mmol) are added. The reaction mixture is stirred between 17 h-72 h. After that, EtOAc (200 mL) is added and the organic phase is extracted, first with 10% HCl and then with saturated NaHCO_3_. The basic phase is treated with HCl 37% until pH 2, usually shown by the color changing to yellow. This phase is extracted with dichloromethane (3 × 75 mL), dried with anhydrous Na_2_SO_4_ and filtered. The solvent is removed *in vacuo*. The resulting residue is precipitated with diethyl ether and filtered in order to obtain a yellow-orange solid. Sometimes, when DMAP is added, the compound precipitates due to its acidity. In those cases, HCl (300 mL) is added and the mixture is stirred gently. The 3-amino-1,4-di-*N*-oxide-quinoxaline-2-carbonitrile derivatives are dissolved, and the precipitated compound is filtered. The compound is first washed with 10% HCl and then with diethyl ether.

*5-Nitrofuran-2-carboxylic acid (3-cyano-1,4-di-N-oxide-quinoxalin-2-yl)-amide* (**25**). Yield 27.8 %; ^1^H-NMR δ ppm: 8.52-8.48 (m, 2H, H_8_+H_5_); 8.14-8.10 (m, 1H, H_7 _); 8.06-8.02 (m,1H, H_6_); 7.91 (d, 1H, H_4’_, *J*_4´-3´ _= 3.9 Hz); 7.85 (d, 1H, H_3’_); IR ν cm^-1^: 3,263 (m, NH); 2,232 (w, C°N); 1,694 (s, C=O); 1,533 (s, NO_2_); 1,357 (s, NO_2_); 1,333 (s, N^+^O^-^); Anal. Calc. for C_14_H_7_N_5_O_6_: C:49.28%; H:2.07%; N:20.52%. Found: C: 48.88%; H: 2.24%; N: 20.70%.

*5-Nitrofuran-2-carboxylic acid (3-cyano-6-methyl-1,4-di-N-oxide-quinoxalin-2-yl)amide* (**26**). Yield 23.5 %; ^1^H-NMR δ ppm: 8.41 (d, 1H, H_8_, *J*_8-7 _= 8.9 Hz); 8.31 (s, 1H, H_5_); 7.95 (d, 1H, H_7_); 7.89 (d, 1H, H_4’_, *J*_4’-3’ _= 3.8 Hz); 7.84 (d, 1H, H_3´_); 2.61 (s, 3H, CH_3_); IR ν cm^-1^: 3,250 (w, NH); 2,236 (w, C°N); 1,701 (s, C=O); 1,527 (s, NO_2_); 1,355 (s, NO_2_); 1,332 (s, N^+^O^-^); Anal. Calc. for C_15_H_9_N_5_O_6_: C: 50.71%; H: 2.55%; N: 19.71%. Found: C: 50.78%; H: 2.61%; N: 19.83%.

*5-Nitrofuran-2-carboxylic acid (3-cyano-6,7-dimethyl-1,4-di-N-oxide-quinoxalin-2-yl)amide* (**27**). Yield 11.3 %; ^1^H-NMR δ ppm: 8.31 (s, 1H, H_8 _); 8.28 (s, 1H, H_5_); 7.91 (d, 1H, H_4’_, *J*_4’‑3’ _= 3.9 Hz); 7.85 (d, 1H, H_3’_); 2.54 (s, 3H, CH_3_); 2.53 (s, 3H, CH_3_); IR ν cm^-1^: 3,173 (m, NH); 2,942 (w, CH_3_); 2,232 (w, C°N); 1,700 (s, C=O); 1,542 (s, NO_2_); 1,349 (s, NO_2_); 1,317 (s, N^+^O^-^); Anal. Calc. for C_16_H_11_N_5_O_6_: C: 52.04%; H: 3.00%; N: 18.96%. Found: C: 52.35%; H: 3.25%; N: 18.61%.

*5-Nitrofuran-2-carboxylic acid (3-cyano-6-methoxy-1,4-di-N-oxide-quinoxalin-2-yl)amide* (**28**). Yield 30.1 %; ^1^H-NMR δ ppm: 8.45 (d, 1H, H_8_, *J*_8-7 _= 9.0 Hz); 7.90 (d, 1H, H_4´_, *J*_4´-3´_ = 3.9 Hz); 7.85 (d, 1H, *H*_3´_); 7.79 (d, 1H, H_5_, *J*_5-7_ = 2.5Hz); 7.74 (dd, 1H, H_ 7_); 4.04 (s, 3H, OCH_3_); IR ν cm^-1^: 3,129 (m, NH); 2,239 (w, C°N); 1,693 (s, C=O); 1,538 (s, NO_2_); 1,388 (s, NO_2_); 1,326 (s, N^+^O^-^); Anal. Calc. for C_15_H_9_N_5_O_7_: C: 48.53 %; H: 2.44 %; N: 18.86%. Found: C: 48.23%; H: 2.41%; N: 18.66%.

*5-Nitrofuran-2-carboxylic acid (6-chloro-3-cyano-1,4-di-N-oxide-quinoxalin-2-yl)amide* (**29**). Yield 16.5 %; ^1^H-NMR δ ppm: 8.49-8.45 (m, 2H, H_8_+H_5_); 8.13 (dd, 1H, H_7_, *J*_7-8 _= 9.2 Hz, *J*_7‑5_ = 2.1 Hz); 7.87 (d, 1H, H_4’_, *J*_4´-3´_ = 3.8 Hz); 7.83 (d, 1H, H_3’_); 5.75 (s, 1H, NH); IR ν cm^-1^: 3,273 (m, NH); 2,238 (w, C°N); 1,711 (s, C=O); 1,537 (s, NO_2_); 1,351 (s, NO_2_); 1,327 (s, N^+^O^-^); 1,077 (m, Ar-Cl); Anal. Calc. for C_14_H_6_ClN_5_O_6_: C: 44.76 %; H: 1.61%; N: 18.64%. Found: C: 44.72%; H: 1.80%; N: 18.53%.

*5-Nitrofuran-2-carboxylic acid (6,7-dichloro-3-cyano-1,4-di-N-oxide-quinoxalin-2-yl)amide* (**30**). Yield 12.4 %; ^1^H-NMR δ ppm: 8.70 (s, 1H, H_8 _); 8.66 (s, 1H, H_5_); 7.87 (d, 1H, H_4’_, *J*_4’‑3’ _= 3.9 Hz); 7.84 (d, 1H, H_3’_); IR ν cm^-1^: 3,263 (m, NH); 2,244 (w, C°N); 1,711 (s, C=O); 1,539 (s, NO_2_); 1,355 (s, NO_2_); 1,330 (s, N^+^O^-^); 1,073 (m, Ar-Cl); Anal. Calc. for C_14_H_5_Cl_2_N_5_O_6_: C: 41.00%; H: 1.23%; N: 17.08%. Found: C: 41.15%; H: 1.30%; N: 17.25%.

*5-Nitrofuran-2-carboxylic acid (6-fluoro-3-cyano-1,4-di-N-oxide-quinoxalin-2-yl)amide* (**31**). Yield 9.9 %; ^1^H-NMR δ ppm: 8.58 (dd, 1H, H_8_, *J*_8-7 _= 9.4 Hz, *J*_8-F _= 5.0 Hz); 8.30 (dd, 1H, H_5_, *J*_5‑F _= 8.7 Hz, *J*_5-7 _= 2.5 Hz); 8.06-8.01 (m, 1H, H_7_); 7.90 (d, 1H, H_4’_, *J*_4’-3’ _= 3.9 Hz); 7.84 (d, 1H, H_3’_); IR ν cm^-1^: 3,237 (m, NH); 2,238 (w, C°N); 1,708 (s, C=O); 1,536 (s, NO_2_); 1,357 (s, NO_2_); 1,331 (s, N^+^O^-^); 1,114 (m, Ar-F); Anal. Calc. for C_14_H_6_FN_5_O_6_: C: 46.81%; H: 1.68%; N: 19.50% Found: C: 46.53%; H: 1.74%; N: 19.55%.

*5-Nitrofuran-2-carboxylic acid (6,7-difluoro-3-cyano-1,4-di-N-oxide-quinoxalin-2-yl)amide* (**32**). Yield 38.6 %; ^1^H-NMR δ ppm: 8.63 (dd, 1H, H_8_, *J*_8-F7 _= 9.9 Hz, *J*_8-F6 _= 7.2 Hz); 8.58 (dd, 1H, H_5_, *J*_5-F6 _= 9.9 Hz, *J*_5-F7 _= 7.3Hz); 7.89 (d, 1H, H_4’_, *J*_4’-3’ _= 3.9 Hz); 7.84 (d, 1H, H_3’_); 5.76 (s, 1H, NH); IR ν cm^-1^: 3,268 (m, NH); 2,244 (w, C°N); 1,711 (s, C=O); 1,534 (s, NO_2_); 1,394 (s, NO_2_); 1,350 (s, N^+^O^-^); 1,106 (m, Ar-F); Anal. Calc. for C_14_H_5_F_2_N_5_O_6_: C: 44.58%; H: 1.34%; N: 18.57%. Found: C: 44.04%; H: 1.34%; N: 18.33%.

*5-Nitrofuran-2-carboxylic acid (7-trifluoromethyl-3-cyano-1,4-di-N-oxide-quinoxalin-2-yl)amide* (**33**). Yield 39.8 %; ^1^H-NMR δ ppm: 8.67 (s, 1H, H_8_); 8.54 (d, 1H, H_5_, *J*_5-6 _= 8.9 Hz); 8.37 (dd, 1H, H_6_, *J*_6-8 _= 1.6 Hz); 7.84 (s, 2H, H_4´_+H_3´_); IR ν cm^-1^: 3,275 (m, NH); 2,244 (w, C°N); 1,707 (s, C=O); 1,536 (s, NO_2_); 1,391 (s, NO_2_); 1,350 (s, N^+^O^-^); 1,173 (m, Ar-CF_3_); Anal. Calc. for C_15_H_6_F_3_N_5_O_6_: C: 44.02%; H: 1.48%; N: 17.11%. Found: C: 44.08%; H: 1.47%; N: 16.71%.

*5-Nitrofuran-2-carboxylic acid (6-trifluoromethyl-3-cyano-1,4-di-N-oxide-quinoxalin-2-yl)amide* (**34**). Yield 51.8 %; ^1^H-NMR δ ppm: 8.68 (s, 1H, H_5_); 8.66 (d, 1H, H_8_, *J*_8-7_ = 9.1Hz); 8.30 (d, 1H, H_7 _); 7.89 (d, 1H, H_4’_, *J*_4´-3´_ = 3.9Hz); 7.84 (d, 1H, H_3’_). IR ν cm^-1^: 3277 (m, NH); 2238 (w, C°N); 1709 (s, C=O); 1538 (s, NO_2_); 1391 (s, NO_2_); 1350 (s, N^+^O^-^); 1173 (m, Ar-CF_3 _). Calculated analysis for C_15_H_6_F_3_N_5_O_6_: C:44.02%; H:1.48%; N:17.11%. Found: C:44.26%; H:1.61%; N:16.43%.

*5-Nitrothiophene-2-carboxylic acid (3-cyano-1,4-di-N-oxide-quinoxalin-2-yl)amide* (**35**). Yield 36.2 %; ^1^H-NMR δ ppm: 8.49 (s, 1H, H_8 _), 8.47 (s, 1H, H_5_), 8.26-8.23 (m, 2H, H_4’_+H_3´_) 8.11 (dd, 1H, H_6_, *J_6-5 _*= 8.3 Hz, *J_6-7 _*= 7.4 Hz), 8.00 (dd, 1H, H_7_, *J_7-8 _*= 8.1 Hz); IR ν cm^-1^: 3,278 (w, NH); 2,232 (w, C°N); 1,673 (s, C=O); 1,538 (s, NO_2_); 1,356 (m, NO_2_); 1,333 (s, N^+^O^-^); Anal. Calc. for C_14_H_7_N_5_O_5_S: C: 47.06%; H: 1.97%; N: 19.60%. Found: C: 47,16 %; H: 2.31%; N: 19.31%.

*5-Nitrothiophene-carboxylic acid (3-cyano-6-methyl-1,4-di-N-oxide-quinoxalin-2-yl)amide* (**36**). Yield 41.3 % ^1^H-NMR δ ppm: 8.41 (d, 1H, H_8_, *J*_8-7 _= 8.8 Hz); 8.31 (s, 1H, H_5_); 8.25 (s, 2H, H_3’_+H_4’_); 7.96 (dd, 1H, H_7_, *J*_7-8 _= 8.8 Hz, *J*_7-5 _= 1.7 Hz); 2.61 (s, 3H, CH_3_); IR ν cm^-1^: 3,231 (w, NH); 2,238 (w, C°N); 1,677 (s, C=O); 1,528 (m, NO_2_); 1,348 (m, NO_2_); 1,325 (s, N^+^O^-^); Anal. Calc. for C_15_H_9_N_5_O_5_S: C: 48.52%; H: 2.44%; N: 18.86%. Found: C: 48.26%; H: 2.48%; N: 19.05%. 

*5-Nitrothiophene-2-carboxylic acid (3-cyano-6,7-dimethyl-1,4-di-N-oxide-quinoxalin-2-yl)amide* (**37**). Yield 8.6 %; ^1^H-NMR δ ppm: 8.29 (s, 1H, H_8 _); 8.28 (s, 1H, H_5_); 8.24 (s, 2H, H_3´_+H_4´_); 5.76 (s, 1H, NH); 2.54 (s, 3H, CH_3_); 2.52 (s, 3H, CH_3_). IR ν cm^-1^: 3,244 (w, NH); 2,238 (w, C°N); 1,676 (m, C=O); 1,528 (s, NO_2_); 1,372 (m, NO_2_); 1,332 (s, N^+^O^-^). Anal. Calc. for C_16_H_11_N_5_O_5_S: C: 49.87%; H: 2.88%; N:18.17%. Found: C: 49.82%; H: 2.98%; N: 18.00%.

*5-Nitrothiophene-2-carboxylic acid (3-cyano-6-methoxy-1,4-di-N-oxide-quinoxalin-2-yl)amide* (**38**). Yield 5.6 %; ^1^H-NMR δ ppm: 8.43 (d, 1H, H_8_, *J*_8-7 _= 8.8 Hz); 8.22 (d, 1H, H_4´_, *J*_4´-3´ _= 4.0 Hz); 8.16 (d, 1H, H_3´_); 7.77 (s, 1H, H_5_); 7.72 (d, 1H, H_7 _); 4.03 (s, 3H, OCH_3_); IR ν cm^-1^: 3,270 (w, NH); 2,238 (w, C°N); 1,681 (w, C=O); 1,515 (s, NO_2_); 1,348 (m, NO_2_); 1,333 (s, N^+^O^-^); Anal. Calc. for C_15_H_9_N_5_O_6_S: C: 46.51 %; H: 2.34 %; N: 18.08%. Found: C: 46.64%; H: 2.52%; N: 18.28%.

*5-Nitrothiophene-2-carboxylic acid (6-chloro-3-cyano-1,4-di-N-oxide-quinoxalin-2-yl)amide* (**39**). Yield 36.7 %; ^1^H-NMR δ ppm: 8.50 (d, 1H, H_5_, *J*_5-7 _= 2.2 Hz); 8.48 (d, 1H, H_8_, *J*_8-7 _= 9.2 Hz); 8.24 (d, 1H, H_4´_, *J*_4´-3´ _= 4.4 Hz); 8.23 (d, 1H, H_3´_); 8.14 (dd, 1H, H_7_, *J*_7-8 _= 9.2 Hz, *J*_7-5 _= 2.2 Hz); IR ν cm^-1^: 3,275 (w, NH); 2,239 (w, C°N); 1,677 (s, C=O); 1,519 (s, NO_2_); 1,357 (m, NO_2_); 1,323 (s, N^+^O^-^); 1,090 (m, Ar-Cl); Anal. Calc. for C_14_H_6_ClN_5_O_5_S: C: 42.92 %; H: 1.54%; N: 17.88%. Found: C: 43.01%; H: 1.71%; N: 18.02%.

*5-Nitrothiophene-2-carboxylic acid (6,7-dichloro-3-cyano-1,4-di-N-oxide-quinoxalin-2-yl)amide* (**40**). Yield 4.4 %; ^1^H-NMR δ ppm: 8.66 (s, 1H, H_8_); 8.60 (s, 1H, H_5 _); 8.19 (s, 1H, H_4’_); 8.05 (s, 1H, H_3’_); IR ν cm^-1^: 3,256 (w, NH); 2,238 (w, C°N); 1,675 (m, C=O); 1,505 (s, NO_2_); 1,354 (m, NO_2_); 1,330 (s, N^+^O^-^); Anal. Calc. for C_14_H_5_Cl_2_N_5_O_5_S: C: 39.46%; H: 1.18%; N: 16.43%. Found: C: 39.00%; H: 1.35%; N: 16.07%.

*5-Nitrothiophene-2-carboxylic acid (6-fluoro-3-cyano-1,4-di-N-oxide-quinoxalin-2-yl)amide* (**41**). Yield 24.0 %; ^1^H-NMR δ ppm: 8.60-8.56 (m, 1H, H_8_); 8.32-8.29 (m, 1H, H_5_); 8.25 (s, 2H, H_4´_+H_3´_); 8.07-8.02 (m, 1H, H_6_); 5.76 (s, 1H, NH); IR ν cm^-1^: 3,293 (m, NH); 2,238 (w, C°N); 1,671 (s, C=O); 1,514 (s, NO_2_); 1,349 (m, NO_2_); 1,332 (s, N^+^O^-^); 1,114 (m, Ar-F); Anal. Calc. for C_14_H_6_FN_5_O_5_S: C: 44.81%; H: 1.61%; N: 18.66%. Found: C: 44.31%; H: 1.69%; N: 18.44%.

*5-Nitrothiophene-2-carboxylic acid (6,7-difluoro-3-cyano-1,4-di-N-oxide-quinoxalin-2-yl)amide* (**42**). Yield 33.4 %; ^1^H-NMR δ ppm: 8.65-8.55 (m, 2H, H_8_+H_5_); 8.24 (d, 1H, H_4´_, *J*_4´-3´ _= 4.5 Hz); 8.23 (d, 1H, H_3´_); IR ν cm^-1^: 3,256 (w, NH); 2,232 (w, C°N); 1,685 (s, C=O); 1,517 (s, NO_2_); 1,359 (s, NO_2_); 1,341 (s, N^+^O^-^); Anal. Calc. for C_14_H_5_F_2_N_5_O_5_S: C: 42.76%; H: 1.28%; N: 17.81%. Found: C: 42.78%; H: 1.30%; N: 17.89%.

*5-Nitrothiophene-2-carboxylic acid (7-trifluoromethyl-3-cyano-1,4-di-N-oxide-quinoxalin-2-yl)amide* (**43**). Yield 51.8 %; ^1^H-NMR δ ppm: 8.65 (d, 1H, H_5_, *J*_5-6 _= 8.8 Hz); 8.65 (s, 1H, H_8 _); 8.27 (dd, 1H, H_6_, *J*_6-8 _= 1.5Hz); 8.23 (d, 1H, H_4’_, *J*_4´-3´ _= 4.4 Hz); 8.18 (d, 1H, H_3’_); IR ν cm^-1^: 3,244 (w, NH); 2,238 (w, C°N); 1,672 (s, C=O); 1,506 (s, NO_2_); 1,399 (s, NO_2_); 1,338 (s, N^+^O^-^); 1,130 (m, Ar‑CF_3_); Anal. Calc. for C_15_H_6_F_3_N_5_O_5_S: C: 42.36%; H: 1.42%; N: 16.47%. Found: C: 42.58%; H: 1.60%; N: 16.49%.

*5-Nitrothiophene-2-carboxylic acid (6-trifluoromethyl-3-cyano-1,4-di-N-oxide-quinoxalin-2-yl)amide* (**44**). Yield 57.9 %; ^1^H-NMR δ ppm: 8.64 (s, 1H, H_5_); 8.47 (d, 1H, H_8_, *J*_8-7 _= 8.9 Hz); 8.35 (d, 1H, H_7_); 8.20 (d, 1H, H_4’_, *J*_4´-3´ _= 4.3 Hz); 8.08 (d, 1H, H_3’_); IR ν cm^-1^: 3,247 (w, NH); 2,238 (w, C°N); 1,675 (s, C=O); 1,520 (s, NO_2_); 1,399 (m, NO_2_); 1,333 (s, N^+^O^-^); 1,139 (m, Ar-CF_3_); Anal. Calc. for C_15_H_6_F_3_N_5_O_5_S: C: 42.36%; H: 1.42%; N: 16.47%. Found: C: 42.62%; H: 1.72%; N: 16.57%.

### In vitro evaluation of antituberculosis activity

*In vitro* evaluation of the antituberculosis activity was carried out at the GWL Hansen’s Disease Center within the Tuberculosis Antimicrobial Acquisition & Coordinating Facility (TAACF) screening program for the discovery of novel drugs for the treatment of tuberculosis. The Southern Research Institute coordinates the overall program under the direction of the U.S. National Institute of Allergy and Infectious Disease (NIAID). The purpose of the screening program is to provide a resource whereby new experimental compounds can be tested for their capability to inhibit the growth of virulent *M. tuberculosis* [26].

*Determination of growth inhibition percentage via MABA*: The initial screen is conducted against *Mycobacterium tuberculosis* H37Rv (ATCC 27294) in BACTEC 12B medium using the Microplate Alamar Blue Assay (MABA) [27]. The fluorescence changes due to the reduction of Alamar blue dye during the growth of *Mycobacterium* were monitored by the BACTEC 460-radiometric system. Compounds effecting <90% inhibition in the primary screen (MIC >6.25 μg/mL) were not further evaluated. 

*Determination of minimum inhibitory concentration (MIC) via MABA*: Compounds demonstrating at least 90% inhibition in the primary screen were re-tested against *M. tuberculosis* H37Rv at lower concentrations in order to determine the actual minimum inhibitory concentration (MIC) in the MABA. The MIC was defined as the lowest concentration effecting a reduction in fluorescence of 90% relative to controls. RIF was used as the reference compound (RIF MIC = 0.015*20130.125 μg/mL).

### In vitro evaluation of trypanocidal activity

*Trypanosoma cruzi* epimastigotes (Tulahuen 2 strain) were grown at 28 ºC in an axenic medium (BHI-Tryptose) as previously described [28,29,30], supplemented with 5% fetal bovine serum (FBS). Cells from a 10-day-old culture (stationary phase) were inoculated into 50 mL of fresh culture medium in order to give an initial concentration of 1 × 10^6^ cells/mL. Cell growth was followed by measuring the absorbance of the culture at 600 nm daily. Before inoculation, the medium was supplemented with the indicated amount of the drug from a stock solution in DMSO. The final concentration of DMSO in the culture medium never exceeded 0.4%, and the control was run in the presence of 0.4% DMSO and in the absence of any drug. No effect on epimastigote growth was observed by the presence of up to 1% DMSO in the culture medium. The percentage of growth inhibition (PGI) was calculated as follows: PGI (%) = {1 - [(Ap - A0p)/(Ac - A0c)]} × 100, where Ap = A_600_ of the culture containing the drug at day 5; A0p = A_600_ of the culture containing the drug just after addition of the inocula (day 0);Ac = A_600_ of the culture in the absence of the drug (control) at day 5; A0c = A_600_ in the absence of the drug at day 0. In order to determine IC_50_ values, 50% inhibitory concentrations, parasite growth was observed in the absence (control) and presence of increasing concentrations of the corresponding drug. At day 5, the absorbance of the culture was measured and related to the control. The IC_50_ value was considered to be the concentration of drug needed for reducing the absorbance ratio to 50%.

## Conclusions

New structural modifications on the QDO skeleton were performed, and promising biological results against *M. tuberculosis* and *T. cruzi* were obtained. The biological evaluation showed a broad range of activities, thereby showing new structural hits for future chemical pharmacomodulations of QDO for the development of new drugs against tuberculosis and Chagas disease.
